# Multi-Hop Localization Algorithm Based on Grid-Scanning for Wireless Sensor Networks*

**DOI:** 10.3390/s110403908

**Published:** 2011-03-31

**Authors:** Jiangwen Wan, Xiaolei Guo, Ning Yu, Yinfeng Wu, Renjian Feng

**Affiliations:** School of Instrument Science and Opto-Electronics Engineering, Beijing University of Aeronautics and Astronautics (Beihang University), Beijing 100191, China; E-Mails: jwwan@buaa.edu.cn (J.W.); xiaoleiguo@aspe.buaa.edu.cn (X.G.); yfwu@buaa.edu.cn (Y.W.); rjfeng@buaa.edu.cn (R.F.)

**Keywords:** wireless sensor networks, multi-hop localization, feasible region, grid-scanning

## Abstract

For large-scale wireless sensor networks (WSNs) with a minority of anchor nodes, multi-hop localization is a popular scheme for determining the geographical positions of the normal nodes. However, in practice existing multi-hop localization methods suffer from various kinds of problems, such as poor adaptability to irregular topology, high computational complexity, low positioning accuracy, *etc.* To address these issues in this paper, we propose a novel *Multi-hop Localization algorithm based on Grid-Scanning* (MLGS). First, the factors that influence the multi-hop distance estimation are studied and a more realistic multi-hop localization model is constructed. Then, the feasible regions of the normal nodes are determined according to the intersection of bounding square rings. Finally, a verifiably good approximation scheme based on grid-scanning is developed to estimate the coordinates of the normal nodes. Additionally, the positioning accuracy of the normal nodes can be improved through neighbors’ collaboration. Extensive simulations are performed in isotropic and anisotropic networks. The comparisons with some typical algorithms of node localization confirm the effectiveness and efficiency of our algorithm.

## 1. Introduction

Recent advances in the fields of wireless communication, micro-electro-mechanical systems (MEMS) and embedded processing have enabled the emergence of wireless sensor networks (WSNs). WSNs consist of a large number of low-cost, low-consumption, small-size, and multi-functional sensor nodes. Usually, they are randomly deployed (e.g., nodes are scattered from the air) in complex environments to execute a wide variety of tasks, such as environmental monitoring, bush fire surveillance, wildlife behavior studies, target tracking, battlefield spying, *etc.* ([1–3]). For these purposes, each sensor node needs to collaborate with others in sensing events of interest by exchanging acquired data. If the data sent by a node carries no or incorrect position information, they would be meaningless or even harmful. In addition, the positions of sensor nodes are in great demand for some location-aware network protocols, such as location-based routing, data aggregation, node querying, *etc.* Therefore, node localization is an important subject in WSNs. In recent years, various node localization schemes for WSNs have been proposed and a comprehensive survey is provided in [4–6] and the references therein.

The task of WSN node localization is to determine the positions of sensor nodes without initial location information (normal or unknown nodes) based on the knowledge of sensor nodes with initial location information (anchor or beacon nodes) and inter-node distance or bearing measurements. Since anchor nodes usually obtain their coordinates from global positioning system (GPS) receivers or manual configuration in fixed places, raising the number of anchor nodes will significantly increase the cost of network deployment. They should therefore make up only a small proportion of nodes in large-scale WSNs. Thus, many normal nodes may fail to estimate their positions due to their short-range measurement. To solve this problem, three types of localization schemes are proposed, namely, centralized algorithms, recursive algorithms, and multi-hop algorithms.

In centralized algorithms, a powerful processing node collects all inter-node measurements to produce a global topology map of the WSN and then distributes all the nodes’ location information to the network. Typical centralized algorithms include MDS-MAP [7], SDP [8], SA [9], *etc.* Centralized algorithms are likely to provide more accurate location estimates than others, but they are less energy-efficient. This is because shuttling every node’s measurement data to the central node would bring about high energy consumption and put too high a strain on nodes that are close to the central node. In addition, centralized algorithms have poor scalability and generally are not suitable for application in large-scale WSNs. Contrary to centralized algorithms, recursive and multi-hop algorithms are two distributed localization technologies. In recursive algorithms, the localization process propagates from an area that is close to the initial anchor nodes to an area where the initial anchor nodes are inaccessible. Any normal node that has estimated its position becomes a secondary anchor node, and broadcasts its coordinates to assist other nodes in estimating their locations. Recursive algorithms perform well in small-scale networks, but in large-scale WSNs, they suffer from the adverse effects of error propagation and accumulation. With the increased number of iterations, the localization errors would be progressively transmitted and amplified, eventually leading to unbounded errors. In multi-hop algorithms, the normal nodes are not necessarily the one-hop neighbors of anchor nodes. At any time, each node only exchanges its available estimates to anchor nodes acquired so far with its immediate neighbors. Based on the local information collected from neighbors, most normal nodes could be localized simultaneously. Multi-hop algorithms could prevent the propagation of localization errors. They have better real-time performance and require less communication overhead. Therefore, multi-hop localization has received more and more attention in recent years.

In this paper, we analyze the advantages and disadvantages of existing node localization schemes and propose a novel *Multi-hop Localization algorithm based on Grid-Scanning* (MLGS) for large-scale WSNs [10]. Our contributions can be summarized as follows:

1. To improve the topology adaptability and accuracy of multi-hop localization, we study the factors that influence the multi-hop distance estimation and give a quantitative rule for setting the weight of reference information, based on which a more realistic weighted constrained multi-hop localization model is constructed.
2. We come up with a novel approach to determine the scope of node coordinates. Due to the uncertainties in estimated distances, the normal nodes could not be localized in fixed points accurately. Usually, they could only be bounded in a certain region. In this paper, we define the feasible region as the intersection of bounding square rings. By computing the feasible region, we are able to restrict the candidates of node coordinates within a small scope.
3. We design a lightweight and local optimum-avoidable method for the estimation and refinement of node coordinates based on grid-scanning, which is very suitable to senor nodes of limited energy and computing power. Extensive simulations show that MLGS has higher localization accuracy and less computation cost than existing typical schemes, and can perform well, even in anisotropic networks.

The remainder of the article is organized as follows. Section 2 discusses some of the previous works on WSN node localization. Section 3 formulates the multi-hop localization problems and introduces the necessary definitions. Section 4 presents in detailed the MLGS algorithm procedure. Section 5 evaluates the performance of MLGS through experiments. Finally, Section 6 concludes this paper.

## 2. Related Works

### 2.1. Centralized Algorithms

In the literature, there exist three main kinds of centralized localization algorithms [5]: multidimensional scaling (MDS), convex programming and stochastic optimization approaches. Shang *et al.* [7] proposed a centralized algorithm called MDS-MAP. By applying MDS technology to the matrix comprised by the distances or hop counts between all pairs of nodes, the relative positions of all nodes can be obtained. MDS-MAP is robust to measurement errors and only needs a small number of anchor nodes (three or more for 2D, four or more for 3D) to estimate the absolute coordinates of normal nodes. For a network that has *n* nodes, MDS-MAP needs to take *O*(*n*^3^) operations to compute all nodes’ coordinates. With the increase of network size, the operations of MDS-MAP increase dramatically. To make MDS-MAP more applicable to WSNs and have a better performance in irregularly-shaped networks, Shang *et al.* [11] improved MDS-MAP to a distributed fashion by using patches of relative maps, namely, MDS-MAP(P). The main idea of MDS-MAP(P) is to build a local map at each node of the immediate vicinity and then merge these maps together to form a global map. Since a large number of iterations are required for MDS-MAP(P) to converge, the communication and computation cost in map-merging process is high.

Doherty *et al.* [12] modeled the peer-to-peer communication of all nodes as a set of geometric constraints and yielded the global solutions of all unknown coordinates using convex optimization technology. They also gave a method for placing rectangular bounds around the possible positions for all normal nodes. Providing that the constraints are tight enough, the estimated values of this scheme are close to the actual positions of normal nodes. However, when the network density is small or the anchor nodes are not placed around the network boundary, the performance of this scheme would decrease significantly. Biswas *et al.* [8] formulated WSNs localization as a semi-definite programming (SDP) problem through relaxation. The optimization problem is set up so as to minimize the errors in sensor positions for fitting the distance measurements. Similar to MDS-MAP, SDP requires only a few anchor nodes to estimate the positions of all normal nodes in WSNs, but it still demands extensive storage and computation when the network size is large.

To solve the problem of flip ambiguity in WSNs localization, Kannan *et al.* [9] proposed a two-phase localization (SAL) algorithm based on simulated annealing, and it is still a centralized algorithm. Simulated annealing is a stochastic optimization technique that is robust against being trapped in local minima. In the first phase of SAL, simulated annealing is used to obtain the initial location estimation. Then, a second phase of optimization is performed only on those nodes that are likely to have flip ambiguity problems based on the neighborhood information of nodes. SAL gives better accuracy than SDP and does not propagate localization errors, but SAL may fail to identify the flipped node when the network density is low, and the computation and communication cost of SAL is higher.

### 2.2. Iterative Algorithms

Iterative localization schemes, such as the *ad hoc* localization system (AHLoS) [13], usually have a three-phase process. In the first phase, the normal nodes estimate the distances to their neighboring anchor nodes. In the second phase, the normal nodes compute their coordinates using the ranging information and the positions of their neighboring anchor nodes. In the third phase, any normal node that has estimated its position becomes an anchor node and assists other nodes in calculating their coordinates. This process iterates to estimate the positions of as many nodes as possible. Although iterative algorithms only need a small number of clustered anchors to localize the majority of normal nodes, they suffer from the propagation and accumulation of localization errors, especially in large-scale WSNs.

Most recent research works on iterative localization are focus on how to minimize the jeopardy of accumulated errors. Liu *et al.* [14] studied some questions such as where localization error comes from and how it propagates from a node to another one, and then developed an error control mechanism based on the characterization of node uncertainty and the active selection strategy of anchor nodes. The error control mechanism uses only local knowledge and can mitigate the effect of error propagation for both range and directional sensors to a certain extent. Yu *et al.* [15] proposed a two-stage localization scheme. First, localization starts from the nodes with the largest numbers of neighboring anchors and priority is always given to nodes with more neighboring anchors or localized nodes. Then, the locations of all neighboring nodes are exploited to improve localization accuracy. During the localization process, a number of measures are also taken to ensure the reliability of each location estimate to avoid abnormal errors and reduce error propagation. Vemula *et al.* [16] formulated the sensor localization from a probabilistic point of view and proposed four schemes that incorporate anchor position uncertainty to estimate the distribution (mean and covariance) of node coordinates, including iterative least squares (LS) and Bayesian (BS) methods, Monte Carlo importance sampling (IS) and cost-based (CS) methods. These schemes above have relatively good performance in inhibiting the accumulation of localization errors, but the high computational complexity and increased communication cost limit their application in practice.

### 2.3. Multi-Hop Algorithms

By approximating the length of the shortest path to the Euclidean distance, multi-hop localization schemes can infer the distances between any pairs of non-neighboring nodes. Based on the idea of Distance Vector (DV) routing and GPS positioning, Niculescu *et al.* [17] proposed DV-distance (range-based) and DV-hop (range-free) algorithms. They are the origination of multi-hop localization schemes for WSNs. In both algorithms, each anchor node first broadcasts a message that carries its location information to its immediate neighbors. Then, the message is propagated in WSNs in a controlled flood manner that is similar with the distance vector routing. At the same time, each normal node estimates the lengths of shortest paths or minimum hop counts to anchor nodes. If a normal node obtains the estimates to at least three (for 2D) or four (for 3D) anchor nodes, its position can be calculated by using multilateration. DV-distance and DV-hop are low-cost localization solutions, but their accuracy is built on the assumption that the shortest path between a pair of nodes is close to a straight line, which may not always be achievable in anisotropic or sparse networks.

Lim *et al.* [18] designed a proximity-distance map (PDM) to characterize the anisotropic features of WSNs. Actually, PDM is a semi-centralized algorithm. First, the anchor nodes derive an optimal linear transformation collaboratively to map the precise Euclidean distances and the proximities between pairwise anchors. Then, the map is sent to normal nodes to assist them in modifying their multi-hop distance estimations. The intuition of PDM is that the topology character of entire WSNs can be well represented by anchor nodes, but it is not the case in anchor clustered networks. Cheng *et al.* [19] investigated the effect of adverse placement and density of anchors on the accuracies of different algorithms, and developed an algorithm called hybrid localization (HyBloc) to provide reliable localization service with a limited number of clustered anchors. HyBloc combines two techniques, MDS-MAP and PDM. First, MDS-MAP is used to increase the number of anchor nodes in order to extend the anchor coverage of PDM. Then, the normal nodes are localized through PDM. HyBloc could give results as accurate as those of MDS-MAP and is less susceptible to the adverse effect of anchor placement, but it requires more communication and computation cost than PDM.

Shang *et al.* [20] studied the effect of anchor selection on multi-hop localization of WSNs. The experimental results show that using only the four nearest anchor nodes could get better localization performance in most cases. In the rest of this paper, we denote this algorithm as 4-Multihop. Wong *et al.* [21] proposed a density-aware hop-count localization (DHL) algorithm. In DHL, node density is considered and an empirical range ratio (the ratio of expected hop distance to node’s transmission range for a given local density) table is constructed to reduce the overestimation of multi-hop distances. Xiao *et al.* [22] proposed a novel scheme called reliable anchor-based localization (RAL) to eliminate the adverse impact of detoured paths from unreliable anchor nodes. Based on the theoretical analysis of the minimum hop length for uniformly distributed networks, a reliable minimal hop-length table that can help to judge whether a multi-hop path is severely detoured is constructed offline. At runtime, each node only utilizes the distance constraints obtained from reliable anchors to determine its position. Wang *et al.* [23] presented an improved multi-hop algorithm called i-Multihop to minimize the effect of erroneous multi-hop estimated distances on node localization. i-Multihop has higher computational complexity. First, the upper bound constraints are used to filter out the incorrect distance estimations and the estimated position is pinpointed to the intersection constrained by the correct distances. Second, the distance fitting is used to fit correct distance measurements, which makes the final estimated position is not affected by the layout of anchor nodes. Wan *et al.* [24] were concerned with the optimization problem for coordinate calculation in node localization and proposed three schemes based on least squares (LS) and multilateration, namely, Taylor-LS, weighted Taylor-based least squares (WLS) and constrained total least squares (CTLS). Moreover, a generalized Cramér-Rao lower bound (CRLB) is developed to theoretically analyze the performance of multi-hop localization approaches. Although these methods above can guarantee localization performance under certain conditions, most of them may expose certain problems in practice, which include: (1) lacking a local or global geometrical view of WSNs, they are vulnerable to irregular network topologies, (2) unreasonable to treat every reference information in a same priority, especially the one-hop information and the multi-hop one, their localization accuracy needs to be improved, (3) high computational complexity and easy to get stuck at local optimum. These problems inspire the work of this paper.

## 3. Preliminaries

### 3.1. Problem Formulation

**Figure 1.**
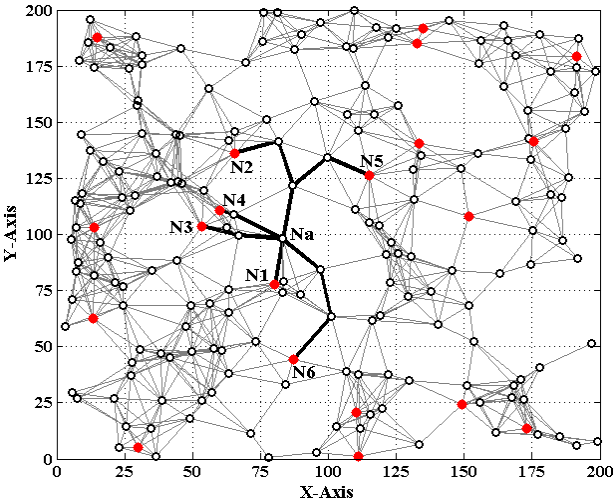
Isotropic network.

**Figure 2.**
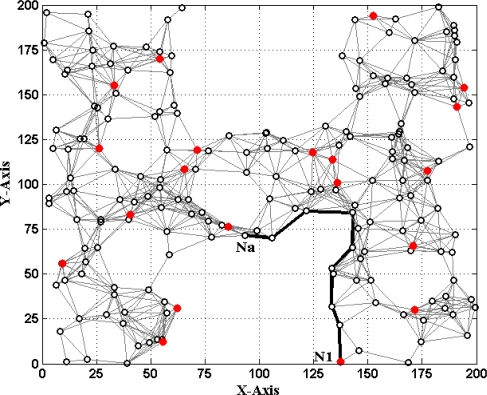
Anisotropic network (H shape).

Figures 1 and 2 show two different types of WSNs. The solid dots ‘ 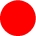
’ and hollow dots ‘○’ represent anchor nodes and normal nodes, respectively. In Figure 1, all nodes are randomly scattered in a 200 × 200 square area to form an isotropic network, while all nodes in Figure 2 are deployed in an H-shaped area to form an anisotropic network. In practice, the anisotropic characteristic results from certain unavoidable reasons, such as non-convex deployment region, node failure or movement, different node densities, obstacle interfering, *etc.* A typical example of anisotropic network is that WSNs are deployed in streets of urban areas where nodes may be separated from each other by buildings, which results in H-shape topology. Without loss of generality, we consider a network consisting of *m* anchor nodes and *n* normal nodes. The identities (IDs) of anchor nodes are from 1 to *m* and those of normal nodes are from *m +* 1 to *m + n*. Each node’s communication and ranging radius is *R*. The network graph can be defined as ***G*** = (***V****_m_* ∪ ***V****_n_*, ***E***), where ***V****_m_* and ***V****_n_* are respectively anchor node set and normal node set. ***E*** is measurable distance set of all pairs of neighboring vertexes (*i*, *j*), *i*, *j* ∈ ***V****_m_* ∪ ***V****_n_*. The objective of WSNs localization is to recover the coordinates of the vertexes in normal node set ***V****_n_* under the constraints of edge set ***E*** and anchor node set ***V****_n_*. The coordinates of node *N_p_* can be described by ***X****_p_* = [*x_p_*, *y_p_*]*^T^*. The Euclidean distance from *N_p_* to its neighbor *N_q_* is *d_pq_* = ‖***X****_p_*–***X****_q_*‖_2_. The corresponding measurable distance is *d′_pq_* = *d_pq_* + *ɛ_pq_*, where the ranging error *ɛ_pq_* ∈ (−*αd_pq_*, *αd_pq_*). The ranging error factor *α* reflects the ranging capability of sensor nodes.

In multi-hop scenarios, through hop by hop dissemination of the estimated distances to anchor nodes in a controlled flooding manner [17], the normal node *N_a_* can estimate the distance *d′_ai_* to the anchor node *N_i_*. *d′_ai_* includes three cases:

1. If *N_a_* and *N_i_* are neighboring nodes, *N_a_* can measure the distance to *N_i_*. Thus *d′_ai_* is the measurable distance between *N_a_* and *N_i_*.
2. If *N_a_* and *N_i_* are non-neighboring nodes, but *N_a_* does not exceed the TTL (time to live) field of *N_i_*’s propagation packets, *d′_ai_* can be approximated by the length of shortest path between *N_a_* and *N_i_*.
3. If *N_a_* exceeds the TTL field of *N_i_*’s packets, *d′_ai_* can’t be estimated by multi-hop information transmission. Thus we denote *d′_ai_* = ∞.

As shown in Figure 1, when *N_a_* gets enough estimated distances *d′_ai_* (*i* = 1, 2, ⋯, *K* to anchor nodes, a system of Euclidean equations can be set up:

(1) $$\{\begin{array}{c} \sqrt{{\left(x_{a}-x_{1}\right)}^{2}+{\left(y_{a}-y_{1}\right)}^{2}}=d_{a1}^{\prime }\\ \sqrt{{\left(x_{a}-x_{2}\right)}^{2}+{\left(y_{a}-y_{2}\right)}^{2}}=d_{a2}^{\prime }\\ \vdots \\ \sqrt{{\left(x_{a}-x_{K}\right)}^{2}+{\left(y_{a}-y_{K}\right)}^{2}}=d_{aK}^{\prime } \end{array}$$

where *K* represents the maximum number of estimated distances *N_a_* gets (in Figure 1, *K* = 6). In 2D scenarios, the node localization problem can be seen as solving an intersection point among several circles. In this case, at least three estimated distances are required to determine a normal node’s position. Similarly, in 3D scenarios, it requires at least four spheres to determine an intersection point, *i.e.*, *K* should be no less than four. In our paper, we mainly consider the 2D localization problem.

If *d′_ai_* is accurate, solving (1) can obtain the true value of *N_a_*’s coordinates ***X****_a_* = [*x_a_*, *y_a_*]*^T^*. However, due to ranging errors and approximations of multi-hop distances, *d′_ai_* suffers from certain uncertainty which directly leads to the localization errors of sensor nodes. Especially in anisotropic networks, the shortest paths between pairs of non-neighboring nodes may be distorted by concave area and deviate far away from the Euclidean distances (e.g., the shortest path *P_a_*_1_ between *N_a_* and *N*_1_ shown in Figure 2). In this case, approximating the lengths of shortest paths to Euclidean distances would give rise to erroneous localization results. How to mitigate the influence of irregular network topology on node localization and improve the localization accuracy is one topic of our study.

Various optimization approaches have been proposed to solve the multilateration problems, among which nonlinear least squares solver (e.g., Levenberg-Marquardt method) and Taylor-series estimator are the most commonly used. However, most of these optimization methods are complex and resource-intensive and therefore usually not applicable to resource-limited sensor nodes. In addition, these methods contain an iterative operation procedure which usually converges to a local minimum close to the initial point. To get a better solution, they need an ideally initial point that is approaching to node’s actual position, but it is not an easy task to obtain such a point. Therefore, reducing the computational complexity and preventing the local optimum from emergence is another main topic of this paper.

### 3.2. Definitions

Before describing our MLGS algorithm, we introduce some necessary definitions:

1. *Local density* (*LD*) [21]: the number of neighboring nodes per node’s communication area. If *N_a_* has *T_a_* neighboring nodes, we denote the local density of *N_a_* as *LD_a_* = *T_a_*. Given a network consisting *n* nodes, its network connectivity is defined as the average value of *n* nodes’ local densities.
2. *Multi-hop density* (*MHD*): if the shortest path *P*_1_*_K_* between node *N*_1_ and *N_K_* passes nodes {*N*_1_, *N*_2_, ⋯, *N_K_*}, the sum of the *K* nodes’ local densities is defined as *P*_1_*_K_*’s multi-hop density. We denote that:
  (2) $${\mathrm{MHD}}_{1K}=\sum_{i=1}^{K}{\mathrm{LD}}_{i}$$
3. *Multi-hop count* (*MHC*): the minimal number of hops between a pair of non-neighboring nodes (also the number of line segments in a shortest path). If the shortest path *P*_1*K*_ passes *K* nodes, *P*_1*K*_’s multi-hop count is *MHC*_1*K*_ = *K* − 1.
4. *Bounding square ring* (***BSR***): the constraint region in the shape of square ring where a normal node is. Based on the estimated distance *d′_ai_*, *N_a_* can obtain one of its bounding square rings, which is denoted as ***BSR****_ai_*.
5. *Feasible region* (***FR***): the intersection area of *N_a_*’s all bounding square rings is defined as *N_a_*’s feasible region. In general, the smaller ***FR****_a_* is, the more accurately *N_a_*’s coordinates can be pinpointed. Therefore, the size of ***FR****_a_* can be regard as the criterion of reckoning the localization accuracy of node *N_a_*.

The details of bounding square ring and feasible region will be discussed in Section 4.3.1.

(6) *Grid granularity* (*g*): a normal node’s feasible region can be divided into some sub-grids of equal size, the size of a sub-grid is called grid granularity. It can be represented by the ratio of side length of a sub-grid to node’s communication radius. The details of grid granularity will be described in Section 4.3.2.

## 4. MLGS Algorithm

In this section, we describe the proposed MLGS algorithm for WSN node localization. In general, MLGS can be divided into four phases: network initialization, construction of multi-hop localization model, estimation of node coordinates, and localization refinement (an optional phase). The details of each phase are given in the following.

### 4.1. Network Initialization

**Figure 3.**
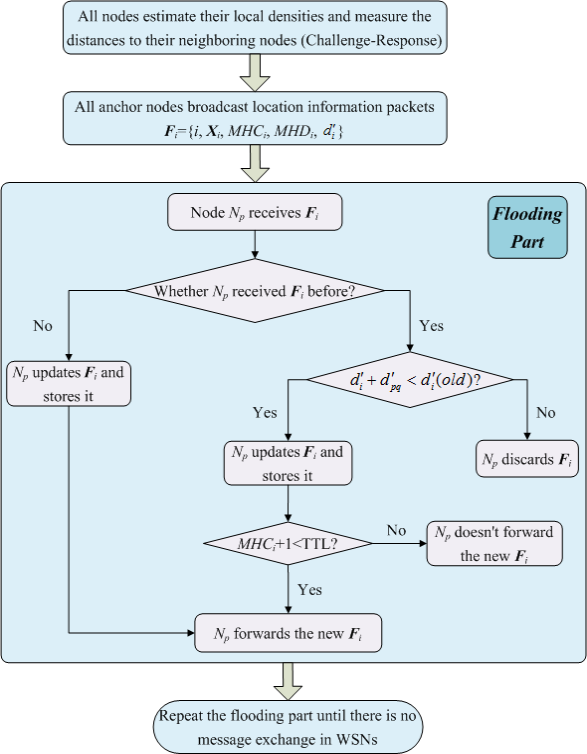
Network initialization procedure.

Similar but not identical to the DHL algorithm proposed by Wong *et al.* [21], in MLGS, the network is initialized in a controlled flood manner that is aware of path-length (in distance) and multi-hop density. We also set a TTL field for propagation packets to reduce the communication cost of sensor nodes. The steps of network initialization are shown as follows (see Figure 3):

***Step*** **1.** Each node first broadcasts a challenge packet ‘I’m *N_p_*. Who is my neighbor?’. Any node that receives the challenge packet then sends a response packet ‘I’m *N_q_*. I’m your neighbor.’ to the corresponding node. All nodes count the number of respond packets they receives to get their local densities. At the same time, all nodes measure the distances to their neighboring nodes.

***Step*** **2.** Each anchor node *N_i_* broadcasts a location information packet ***F****_i_* = {*i*, ***X****_i_*, *MHC_i_*, *MHD_i_*, *d′_i_*} that contains its ID *i* and coordinates ***X****_i_*. Here, *MHD_i_* is multi-hop density of the shortest path to *N_i_*. Its initial value is *N_i_*’s local density *LD_i_*. *MHC_i_* and *d′_i_* are multi-hop count and length of the shortest path to *N_i_*, respectively. Both initial values are set to 0.

***Step*** **3.** When node *N_p_* receives ***F****_i_* that is directly transmitted by *N_i_* (*N_p_* is *N_i_*’s neighboring node), it upgrades ***F****_i_* to {*i*, ***X****_i_*, *MHC_i_* + 1, *MHD_i_* + *LD_p_*, *d′_i_* + *d′_pi_*}, then stores and forwards the new ***F****_i_*.

***Step*** **4.** When node *N_p_* receives ***F****_i_* that is forwarded by its neighboring node *N_q_* (*N_p_* is not *N_i_*’s neighboring node), it first examines whether it received ***F****_i_* before. If not, same to step 3, *N_p_* updates ***F****_i_* to {*i*, ***X****_i_*, *MHC_i_* + 1, *MHD_i_* + *LD_p_*, *d′_i_* + *d′_pq_*}, stores and forwards the new ***F****_i_*. Otherwise, there are two cases:

1. If *d′_i_* + *d′_pq_* < *d′_i_*(*old*), where *d′_i_*(*old*) is the multi-hop estimated distance *N_p_* has stored, *N_p_* updates ***F****_i_* and stores it. When *MHC_i_* + 1 < TTL, *N_p_* forwards the new ***F****_i_*. Otherwise, *N_p_* doesn’t forward it.
2. If *d′_i_* + *d′_pq_* ≥ *d′_i_*(*old*), *N_p_* discards the newly received ***F****_i_*.

***Step*** **5.** Repeat steps 3 and 4 until there is no message exchange in the network. Finally, the normal nodes can get the multi-hop counts, the multi-hop densities and the lengths of shortest paths to the anchor nodes that do not exceed the range of TTL.

### 4.2. Construction of Multi-Hop Localization Model

When normal node *N_a_* gets enough estimated distances *d′_ai_*(*i* = 1, 2, ⋯, *K* to anchor nodes, a multi-hop localization model can be constructed based on the principle of weighted constrained least squares estimator:

(3) $$\begin{array}{l} {\hat{\text{X}}}_{a}^{\left(0\right)}=\underset{{\text{X}}_{a}}{\text{arg min}}\sum_{i=1}^{K}w_{\mathrm{ai}}{\left({\left\|{\text{X}}_{a}-{\text{X}}_{i}\right\|}_{2}-d_{\mathrm{ai}}^{\prime }\right)}^{2}\\ \text{subject to}\;{\text{X}}_{a}\in {\text{FR}}_{a} \end{array}$$

where ${\hat{\text{X}}}_{a}^{\left(0\right)}$ is the estimative value of *N_a_*’s coordinates ***X****_a_*. ***FR****_a_* is the feasible region of *N_a_*. The weight *w_ai_* of reference information {***X***_*i*_, *d′_ai_*} is in inverse proportion to distance estimation error *Δ_ai_*. The bigger *Δ_ai_* is, the smaller *w_ai_* is, *i.e.*, *w_ai_*∝(1/*Δ_ai_*).

We mainly discuss the rules for setting *w_ai_* in this sub-section. How to determine the feasible region ***FR****_a_* will be discussed in Section 4.3.1. Since the multi-hop distance estimation errors mainly arise from the approximations between the lengths of the shortest paths and the Euclidean distances, they are usually larger than the direct ranging errors. Consequently, the multi-hop reference information should be assigned to a smaller weight in multi-hop localization. According to multi-hop count *MHC_ai_*, we set *w_ai_* as follows:

(4) $$w_{\mathrm{ai}}=\{\begin{array}{cc} 1 & \text{if}\;\;{\mathrm{MHC}}_{\mathrm{ai}}=1\\ 0<p\leq 1 & \text{if}\;\;1<{\mathrm{MHC}}_{\mathrm{ai}}\leq \text{TTL}\\ 0 & \text{if}\;\;{\mathrm{MHC}}_{\mathrm{ai}}>\text{TTL} \end{array}$$

When 1 < *MHC_ai_* ≤ TTL, the value of *p* is complex. Each of the shortest paths should be assigned different weights according to the bending degrees of broken lines, *i.e.*, a winding path should have smaller weight than a straight one. In the following, we analyze it in detail.

**Figure 4.**
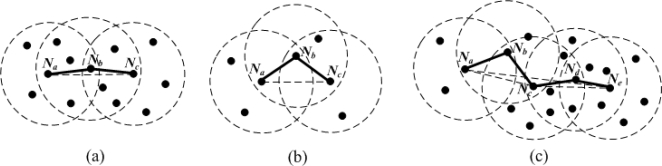
Impact of multi-hop density on multi-hop distance estimation. **(a)** High density. **(b)** Low density. **(c)** High and low density.

Firstly, multi-hop density is an important parameter that affects the multi-hop estimated distance. As can be seen from Figure 4(a), in a dense network, an approximately straight multi-hop path is likely to exist between pairwise nodes.

The length of the shortest path *P_ac_* between *N_a_* and *N_c_* is close to their Euclidean distance. Smaller distance estimation error makes *P_ac_* a higher confidence level. In contrast, if nodes are sparsely deployed [see Figure 4(b)], it is difficult to find a direct multi-hop path between a pair of non-neighboring nodes. The shortest path *P_ac_* is generally more winding than that in Figure 4(a). Figure 4(c) is a combination of the two cases above. Nodes *N_a_*, *N_b_* and *N_c_* have lower local densities, the shortest path *P_ac_* is a winding broken line. However, nodes *N_d_* and *N_e_* have higher local densities, the shortest path *P_ce_* between *N_c_* and *N_e_* is close to a straight line. When we evaluate the bending degree of the shortest path *P_ae_* between *N_a_* and *N_e_*, all nodes’ local densities in *P_ae_* should be considered together. Generally, the larger the multi-hop density is, the more accurate the multi-hop estimated distance is. Therefore, the weight *w_ai_* should be proportional to *MHD_ai_*.

Secondly, with the increase of *MHC_ai_*, the number of line segments in *P_ai_* rises, which reduces the probability that *P_ai_* is close to a straight line. In this case, approximating the length of *P_ai_* to the Euclidean distance *d_ai_* between *N_a_* and *N_i_* would bring larger localization errors. In order to mitigate the influence of the multi-hop distance estimation errors on node localization, we should lower the weight of multi-hop reference information. Therefore, *w_ai_* should be in inverse proportion to *MHC_ai_*. In addition, with the increase of ranging error factor *α* (*i.e.*, nodes’ ranging capability declines), we should appropriately raise the confidence level of multi-hop reference information to weaken the impact of direct ranging results on node localization, since the weight of one-hop reference information remains a constant value of 1 in (4). Based on the analysis above and the results of numerous simulations, the weight *w_ai_* of multi-hop reference information can be set as follows:

(5) $$w_{\mathrm{ai}}=\text{exp}(\alpha )*{\left(\frac{1}{{\mathrm{MHC}}_{\mathrm{ai}}}\right)}^{r}*{\left(\frac{{\mathrm{MHD}}_{\mathrm{ai}}}{({\mathrm{MHC}}_{\mathrm{ai}}+1)*{\mathrm{LD}}_{a}}\right)}^{t}\;\left(r,t=1,2,3,\cdots \right)$$

**Figure 5.**
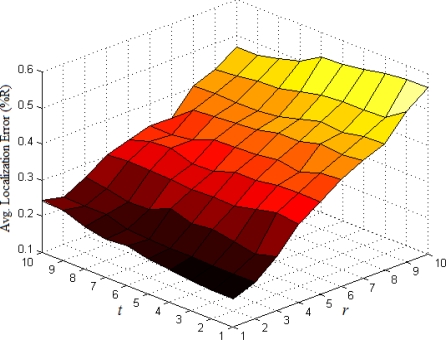
Average localization error as a function of *r* and *t* (isotropic network).

**Figure 6.**
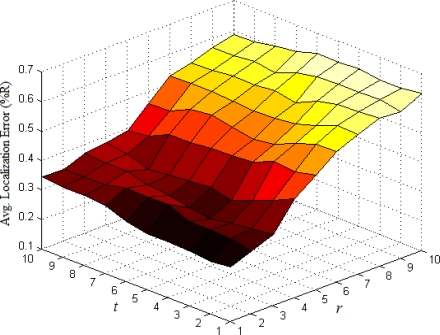
Average localization error as a function of *r* and *t* (anisotropic network).

The base number in the third part of Equation (5) represents the ratio of all nodes’ average density to the target node’s local density. In most cases, Equation (5) could satisfy the requirement that the weight *w_ai_* of multi-hop reference information is no more than 1. If extreme case of *w_ai_* > 1 appears, we set *w_ai_* = 1. To determine the optimal values of indexes *r* and *t*, we have done numerous simulations in various network environments. The results show that we could usually get more ideal localization accuracy when both *r* and *t* are set to 1. Taking the scenarios shown in Figures 1 and 2 as examples, we vary *r* and *t* from 1 to 10 and use classical weighted least squares estimator to compute the coordinates of normal nodes. The average localization errors in both scenarios are shown in Figures 5 and 6, respectively.

The change trend of average errors with varying *r* and *t* in isotropic network is nearly consistent to that in anisotropic network. And the minimum points in both figures usually appear in the lower-left corners of the curved surfaces where both *r* and *t* are equal to 1.

### 4.3. Estimation of Node Coordinates

To simplify the computational complexity of node localization and prevent getting stuck at local optimum, we propose a novel method to estimate the coordinates of normal nodes. First, the feasible regions of normal nodes are determined by calculating the intersection of bounding square rings. Then, the coordinates of normal nodes are estimated through a lightweight grid-scanning procedure. The details of this method are shown as follows.

#### 4.3.1. Determination of Feasible Region

Since the size of feasible regions can reflect the localization accuracy of normal nodes, it is an important task to determine the range of ***FR****_a_* in model (3). In this part, we present how to calculate the feasible regions of normal nodes based on the intersection of bounding square rings. This scheme could restrict the candidates of node coordinates to a small scope.

**Figure 7.**
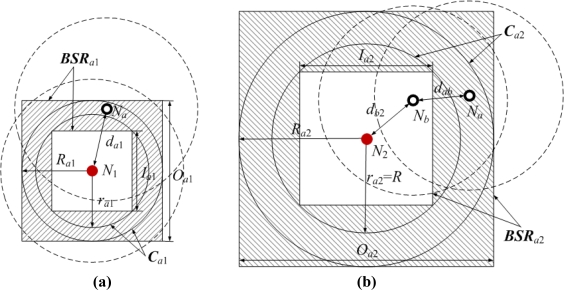
*N_a_*’s bounding square rings. **(a)** Neighboring nodes. **(b)** Non-neighboring nodes.

Figure 7 shows two bounding square rings that are obtained according to the geometrical constraints between pairs of neighboring or non-neighboring nodes. The dashed circles represent sensor nodes’ communication ranges, and the shadow areas represent *N_a_*’s bounding square rings.

In Figure 7(a), normal node *N_a_* and anchor node *N*_1_ are neighboring nodes. *N_a_* can measure the distance to *N*_1_. The measurable distance is denoted as *d′*_*a*1_. As the ranging error *ɛ_a_*_1_ ∈ (−*αd*_*a*1_, *αd*_*a*1_), the Euclidean distance *d_a_*_1_ satisfies the following condition:

(6) $$\frac{d_{a1}^{\prime }}{1+\alpha }\leq d_{a1}\leq \frac{d_{a1}^{\prime }}{1-\alpha }$$

We can infer that *N_a_* is in the circular ring ***C****_a_*_1_ of which the outer radius is *R*_*a*1_ = *d′*_*a*1_ / (1 − *α*) and the inner radius is *r*_*a*1_ = *d′*_*a*1_ / (1 + *α*). The side lengths of the circumscribed and inscribed squares in ***C***_*a*1_ are respectively *O*_*a*1_ = 2*R_a_*_1_ and $I_{a1}=\sqrt{2}r_{a1}$. The shadow area encircled by the circumscribed and inscribed squares is one of *N_a_*’s bounding square rings, and we denote it as:

(7) $${\mathrm{BSR}}_{a1}=\left[x_{1}-\frac{1}{2}O_{a1},x_{1}+\frac{1}{2}O_{a1}\right]\times \left[y_{1}-\frac{1}{2}O_{a1},y_{1}+\frac{1}{2}O_{a1}\right]-\;\left[x_{1}-\frac{1}{2}I_{a1},x_{1}+\frac{1}{2}I_{a1}\right]\times \left[y_{1}-\frac{1}{2}I_{a1},y_{1}+\frac{1}{2}I_{a1}\right]$$

In Figure 7(b), *N_a_* and anchor node *N*_2_ are non-neighboring nodes, but *N_a_* can receive *N*_2_’s location information packet through multi-hop information transmission in the range of TTL. Without loss of generality, we take two hops for example. The shortest path *P_a_*_2_ between *N_a_* and *N*_2_ passes normal node *N_b_*. Since *N_a_* is not in the communication range of *N*_2_, the Euclidean distance *d_a_*_2_ between them is bigger than *R*. Suppose the Euclidean and measurable distance between *N_a_* and *N_b_* are *d_ab_* and *d′_ab_*, and those between *N_b_* and *N*_2_ are *d_b_*_2_ and *d′*_*b*2_. Thus the estimated distance between *N_a_* and *N*_2_ is:

(8) $$d_{a2}^{\prime }=d_{ab}^{\prime }+d_{b2}^{\prime }=(d_{\mathrm{ab}}+{ɛ}_{\mathrm{ab}})+(d_{b2}+{ɛ}_{b2})$$

Based on −*αd_ab_* ≤ *ɛ_ab_* ≤ *αd_ab_* and −*αd_b_*_2_ ≤ *ɛ_b_*_2_ ≤ *αd_b_*_2_, we can infer that:

(9) $$\left(1-\alpha )(d_{\mathrm{ab}}+d_{b2})\leq d_{a2}^{\prime }\leq (1+\alpha )(d_{\mathrm{ab}}+d_{b2}\right)$$

Further:

(10) $$\frac{d_{a2}^{\prime }}{1+\alpha }\leq d_{\mathrm{ab}}+d_{b2}\leq \frac{d_{a2}^{\prime }}{1-\alpha }$$

According to *d_a_*_2_>*R* and *d_a_*_2_≤*d_ab_*+*d_b_*_2_, we have *R* < *d*_*a*2_ ≤ *d′*_*a*2_ / (1 − *α*). Therefore, *N_a_* is also in the circular ring ***C****_a_*_2_ of which the outer radius and inner radius are *R*_*a*2_ = *d′*_*a*2_ / (1 − *α*) and *r_a_*_2_ = *R*, respectively. Using the same method above, we can get *N_a_*’s another bounding square ring ***BSR****_a_*_2_.

**Figure 8.**
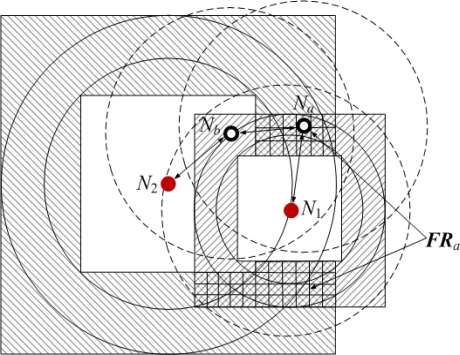
Intersection of bounding square rings.

Figure 8 is the integration of Figure 7(a,b). When *N_a_* gets all its bounding square rings ***BSR****_ai_*(*i* = 1, 2,⋯, *K*), its feasible region ***FR****_a_* (grid area) can be obtained by calculating the intersection of bounding square rings:

(11) $${\mathrm{FR}}_{a}=\overset{K}{\underset{i=1}{\cap }}{\mathrm{BSR}}_{\mathrm{ai}}$$

Details of computing ***FR****_a_* are provided in Appendix A.

#### 4.3.2. Search of Node Coordinates

After obtaining the feasible region ***FR****_a_*, we can employ the classical constrained nonlinear programming solvers, such as sequential quadratic programming (SQP), to work out the optimal value of the objective function in model (3). However, the classical optimization approaches usually involve an iterative computation procedure. In each iterative computation, numbers of complex arithmetical operations (such as matrix inversion, matrix multiplication, eigenvalue determination, *etc.*) are required, which is a severe challenge to the resource-limited sensor nodes. As the number of reference information increases, the computation cost increases dramatically. In addition, the iterative procedure of these methods needs an initial point and easily converge to a local minimum close to the initial point, especially when there is much inaccurate reference information. To solve these problems, a lightweight grid-scanning method is proposed to search the close to optimal values of node coordinates.

Suppose the grid granularity for coordinate estimation is *g*. The value of *g* is determined by some factors, such as range error factor, network connectivity, desired localization accuracy, *etc.* According to *g*, ***FR****_a_* can be divided into a number of sub-grids of equal size (see Figure 8). Regarding the coordinates of all sub-grid’s centers as samples of ***X****_a_*, we can get a sample set:

(12) $$\Omega_{a}=\left\{S_{a}^{\left(1\right)},S_{a}^{\left(2\right)},S_{a}^{\left(3\right)},\cdots ,S_{a}^{\left(U\right)}\right\}$$

where $S_{a}^{\left(i\right)}$ is a sample *of* ***X****_a_* and *U* is the total number of samples. If the area of ***FR****_a_* is *A_a_*, we have *U* = *A_a_*/(*gR*)^2^.

In Appendix A, the computation of feasible region ***FR****_a_* is converted to the problem of calculating the intersection of rectangular sub-regions. As can be seen from Figure B in Appendix, the final output is ***FR****_a_* = ***Array*_*FR****_a_*, where ***Array*_*FR****_a_* is an array consisting of *n* rectangles. This means that the irregular feasible region is divided into several regular rectangles. We further divide each rectangle into sub-grids, so that samples for coordinate estimation can be easily exacted.

After getting ***Ω**_a_*, an optimal sample that brings the objective function in model (3) to the smallest value can be found through scanning ***Ω**_a_* from beginning to end. The final output ${\hat{X}}_{a}^{\left(0\right)}$ of this search procedure is the close to optimal value of ***X****_a_*.

This grid-scanning approach only needs simple arithmetical and comparison operations. It not only has low computational complexity, but also can prevent getting stuck at local optimum. In addition, when the number of reference information increases, it only requires a modest increase in memory consumption and arithmetical operations over those of the classical optimization methods. Therefore, it is a lightweight and efficient method. And it is very suitable to sensor nodes with limited computing and storage capability.

### 4.4. Localization Refinement

After node *N_a_* gets its initial coordinates ${\hat{X}}_{a}^{\left(0\right)}$, it can optionally step into the phase of localization refinement through collaborating with its neighbors. The following are the refinement procedure.

***Step*** **1.** *N_a_* first broadcasts its estimated coordinates and the total number *U* of samples it get in the previous phase. Then, a weighted refinement model for *N_a_* can be constructed based on the broadcast coordinates of *N_a_*’s neighbors and the measurable distances between *N_a_* and its neighbors:

(13) $${\hat{X}}_{a}^{\left(t+1\right)}=\arg\;\underset{X_{a}}{\min}\sum_{b=1}^{J}v_{\mathrm{ab}}{\left({\left\|X_{a}-{\hat{X}}_{b}^{\left(t\right)}\right\|}_{2}-d_{ab}^{\prime (t)}\right)}^{2}$$

where ${\hat{X}}_{b}^{\left(t\right)}(b=1,2,\cdots ,J)$ is the broadcast coordinates of *N_a_*’s neighboring node *N_b_*. *J* is the number of *N_a_*’s neighbors. $d_{\mathrm{ab}}^{\prime (t)}$ is the measurable distance between *N_a_* and *N_b_*. *v_ab_* is the weight of reference information $\left\{{\hat{X}}_{b}^{\left(t\right)},d_{\mathrm{ab}}^{\prime (t)}\right\}$. And *t* is the iteration number of refinement, with an initial value of 0.

Here, we briefly discuss how to set the value of weight *v_ab_*. When *N_b_* is an anchor node, we should set *v_ab_* a bigger value. In contrast, if *N_b_* is a normal node, *v_ab_* should be set a smaller value according to the estimated accuracy of *N_b_*’s coordinates. Since the localization accuracy of normal nodes can be evaluated by the size of feasible regions, *v_ab_* should be in inverse proportion to the total number *U* of samples in 4.3.2. In collaborative refinement, this weighting mechanism not only contributes to the improvement of localization accuracy, but also helps to prevent the localization error from propagation.

**Figure 9.**
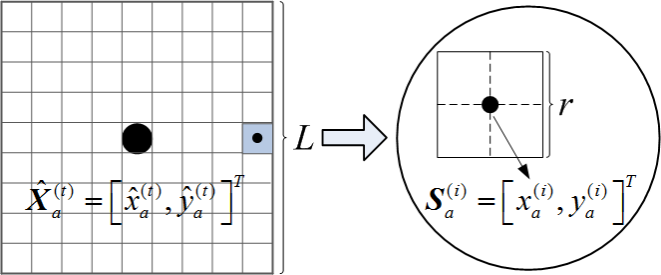
Samples for localization refinement.

***Step*** **2.** The grid-scanning scheme can also be used to seek the close to optimal value of model (13). As can be seen from Figure 9, a square of which the center coordinates and the size length are respectively ${\hat{X}}_{a}^{\left(t\right)}={\left[{\hat{x}}_{a}^{\left(t\right)},{\hat{y}}_{a}^{\left(t\right)}\right]}^{T}$ and *L* (generally no more than *R*) is regarded as *N_a_*’s feasible region for localization refinement, and it is denote as:

(14) $${\mathrm{FR}}_{a}^{\left(t+1\right)}=[{\hat{x}}_{a}^{\left(t\right)}-\frac{1}{2}L,{\hat{x}}_{a}^{\left(t\right)}+\frac{1}{2}L]\times [{\hat{y}}_{a}^{\left(t\right)}-\frac{1}{2}L,{\hat{y}}_{a}^{\left(t\right)}+\frac{1}{2}L]$$

Suppose the grid granularity for localization refinement is *r* (*r* ≤ *g*). According to ${\mathrm{FR}}_{a}^{\left(t+1\right)}$, a sample set ***Θ****_a_* can be obtained by dividing ${\mathrm{FR}}_{a}^{\left(t+1\right)}$ into a number of sub-grids:

(15) $$\Theta_{a}=\left\{S_{a}^{\left(1\right)},S_{a}^{\left(2\right)},S_{a}^{\left(3\right)},\cdots ,S_{a}^{\left(V\right)}\right\}$$

where the number of samples is *V* = *L^2^*/(*rR*)^2^. Through scanning ***Θ****_a_*, the close to optimal value of model (13) can be obtained, denoted as ${\hat{X}}_{a}^{\left(t+1\right)}$. *N_a_* upgrades its estimated coordinates to ${\hat{X}}_{a}^{\left(t+1\right)}$.

***Step*** **3.** *N_a_* broadcasts its new coordinates. Set *t* = *t* + 1. Repeat steps 1 and 2 until the accuracy ( ${\left\|{\hat{X}}_{a}^{\left(t+1\right)}-{\hat{X}}_{a}^{\left(t\right)}\right\|}_{2}\leq r$) is satisfied or the maximum iteration number is reached, whichever comes earlier. The final ${\hat{X}}_{a}^{\left(t+1\right)}$ is the refinement coordinates of *N_a_*.

## 5. Performance Evaluation

In this section, we conduct extensive simulations to study the performance of MLGS algorithm in the isotropic network shown in Figure 1 and the anisotropic network (H-shape) shown in Figure 2. All simulations are run in MatLab R2010a. To reduce the influence of outliers, we run each simulation 100 times and take the average results as the final data points. The default parameters of WSNs are shown in Table 1. Unless specified, we use the default parameters in simulations. We mainly discuss the localization performances of the following four algorithms:

1. The proposed algorithm without refinement phase, denoted as MLGS, in which the grid granularity *g* for coordinate estimation is defaulted as 0.1*R*.
2. The proposed algorithm with refinement phase, denoted as MLGS(R), in which the grid granularity *r* for localization refinement is defaulted as 0.05*R*.
3. The 4-Multihop algorithm proposed by Shang *et al* [20], in which only the four nearest anchor nodes get involved in coordinate estimation. The optimization method employed in 4-Multihop is Taylor-series estimator.
4. The i-Multihop algorithm proposed by Wang *et al.* [23], which combines upper bound and distance consistency, and has higher computational complexity. In i-Multihop, the sequential quadratic programming method is used to solve the constrained nonlinear optimization problem.

### 5.1. Distribution of Node Localization Errors

**Figure 10.**
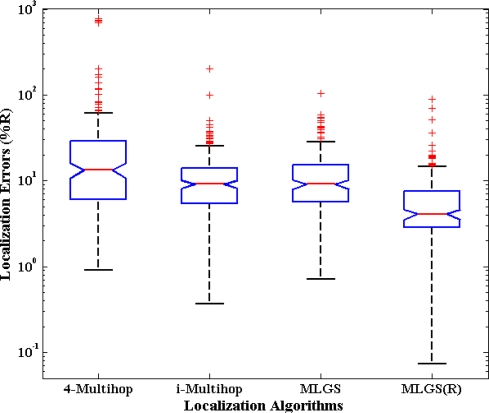
Distribution boxplots of node localization errors (isotropic network).

**Figure 11.**
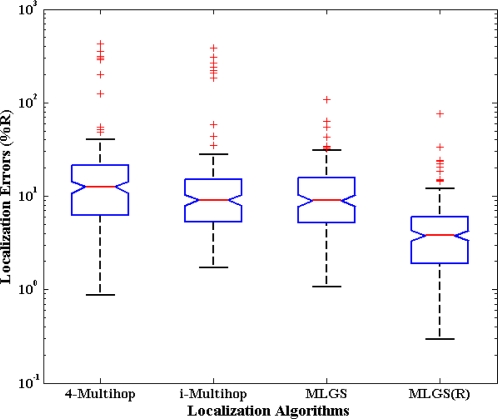
Distribution boxplots of node localization errors (anisotropic network).

First, we analyze the distribution of node localization errors in the default environments. The localization errors are represented by the ratio of the Euclidean distances between estimated coordinates and actual coordinates to node’s communication radius. Figures 10 and 11 present the distribution boxplots for isotropic and anisotropic networks, respectively. The y-axis of both figures is drawn in log-scale. In isotropic network, 4-Multihop gives the worst performance. Its average and median errors are respectively 23.24% and 13.36%, and its maximum outlier is even close to 800%. The average and median errors of i-Multihop are almost the same as those of MLGS. But the errors of i-Multihop are more scattered. The maximum outlier of i-Multihop reaches 200.50% (compared with 103.29% of MLGS). MLGS(R) has an average error of 7.17% and a median error of 4.08%, which is a significant improvement in localization accuracy. In anisotropic network, 4-Multihop is less affected by irregular shape because it only uses the four nearest anchor nodes in calculating the coordinates of normal nodes. Compared with Figure 10, we can see that the accuracy of i-Multihop declines vastly. The average error of i-Multihop reaches 21.67%, but it is still lower than that of 4-Multihop (26.53%). MLGS and MLGS(R) are robust to irregular topologies. Their average errors, median errors and maximum outliers in anisotropic network are almost the same as those in isotropic network, and much smaller than those of 4-Multihop and i-Multihop.

### 5.2. Impact of TTL

**Figure 12.**
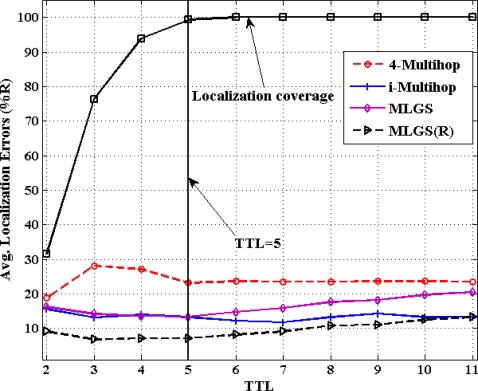
Average localization error *versus* TTL (isotropic network).

**Figure 13.**
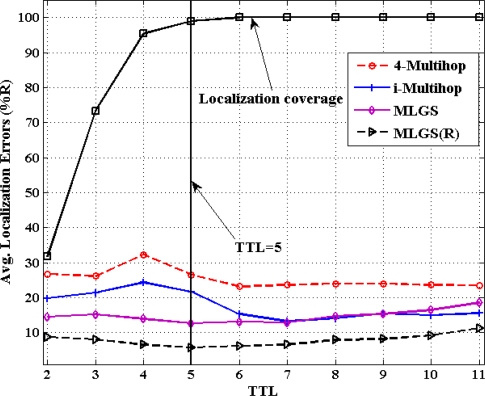
Average localization error *versus* TTL (anisotropic network).

Figures 12 and 13 show the comparison results of average localization errors and localization coverage rates with various TTL. With the increase of TTL, the normal nodes could get more and more reference information for their localization, so the localization coverage rates of four algorithms grow gradually. When TTL reaches 5, the coverage rates in both networks are approaching to 100%. Since raising TTL could increase the communication cost in localization, we try to keep TTL a smaller value in the premise of localizing most nodes. That is the reason why we set the default value of TTL to 5 in simulations.

In isotropic network, 4-Multihop performs the worst. Its average error varies significantly when TTL ≤ 5 and remains generally stable (about 23%) after TTL > 5. The localization accuracies of MLGS, MLGS(R) and i-Multihop are less affected by TTL and are always better than that of 4-Multihop. Among them, MLGS(R) gives the smallest localization error of about 10%. Through the accuracy of i-Multihop slightly exceeds that of MLGS when TTL ≥ 6, but it is always lower than that of MLGS(R). In anisotropic network, 4-Multihop still has the lowest accuracy. i-Multihop is greatly affected by irregular network, and its average error is about 5% higher than that of MLGS when TTL ≤ 5. With the increase of TTL, the number of distance constraints in i-Multihop rises, and the accuracy of i-Multihop is gradually near to that of MLGS. Through refinement, MLGS(R) can increase the localization accuracy by more than 5%. And in most cases, its average error is less than 10% of the communication radius of sensor nodes.

### 5.3. Impact of Network Connectivity

**Figure 14.**
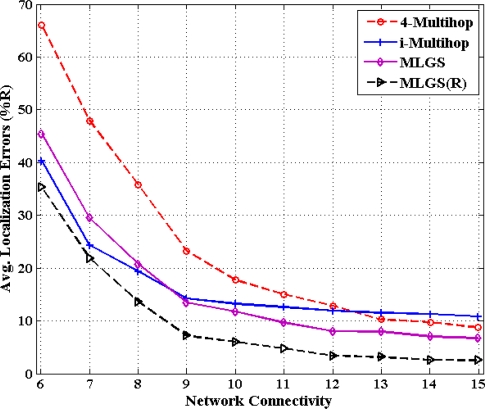
Average Localization error *versus* network connectivity (isotropic network).

**Figure 15.**
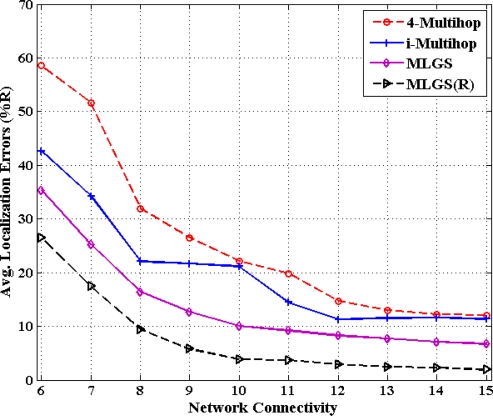
Average localization error *versus* network connectivity (anisotropic network).

In this part, we vary the communication radius of sensor nodes and get the accuracy comparisons of four algorithms under different network connectivity, ranging from 6 to 15 (see Figures 14 and 15). In general, the probability that a shortest path between pairwise nodes is close to a straight line grows as network connectivity increases, which directly results in the improvement of multi-hop localization accuracy. In isotropic network, the performance of MLGS(R) is better than those of three other algorithms. It gives an average error of less than 3.5% for high network connectivity (no less than 12). When network connectivity is smaller than 9, the accuracy of i-Multihop is higher than that of MLGS. However, the average localization error of i-Multihop gradually converges to about 11% and exceeds those of MLGS and 4-Multihop. In anisotropic network, the variation trend of average localization errors of four algorithms is similar to that in isotropic network, but the gap among the four algorithms becomes more evident. The localization accuracies of MLGS and MLGS(R) are always better than those of i-Multihop and 4-Multihop. When network connectivity is 6, the average error of MLGS is less than 40%, above which the localization error will significantly affect the application performance of WSNs [25]. When network connectivity reaches 10, the average error of MLGS is below 10% and it can be further reduced to less than 5% through refinement, while the two other algorithms give larger average errors of more than 20%.

### 5.4. Impact of Ranging Error

**Figure 16.**
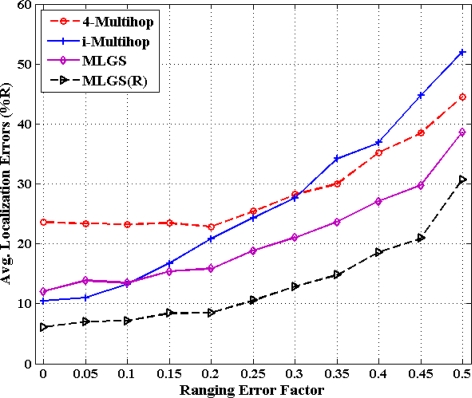
Average localization error *versus* ranging error factor (isotropic network).

**Figure 17.**
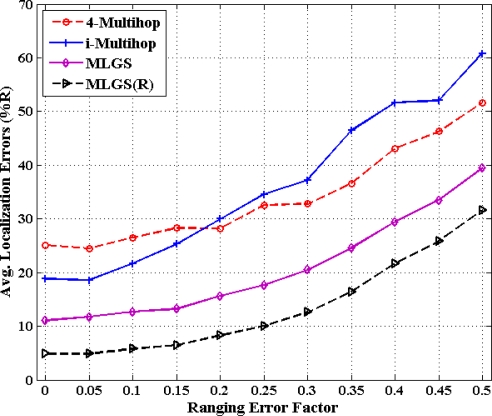
Average localization error *versus* ranging error factor (anisotropic network).

Figures 16 and 17 show the statistics for performance of four algorithms with different ranging error factors. With the increase of ranging errors, the accuracies of four multi-hop algorithms drop gradually. Among them, i-Multihop is the most sensitive to ranging errors. In isotropic network, the average error of i-Multihop is near to 10% when *α* < 0.1, while that of MLGS is about 12%. However, when *α* increases to 0.1, the two algorithms produce similar results. And after that, the average error of i-Multihop increases substantially and even exceeds that of 4-Multihop when *α* = 0.35.

As can be seen from Figure 16, we can infer that MLGS and MLGS(R) are robust with respect to high ranging errors. When *α* increases to 0.5, the average localization error of MLGS is still smaller than 40% of node’s communication radius. Through refinement, the average error can be further reduced to 30%. In anisotropic network, MLGS and MLGS(R) perform consistently better than 4-Multihop and i-Multihop under all ranging error factors considered. Compared with the latter two algorithms, MLGS can improve localization accuracy by 10%∼20%, and further increase by 5% after refinement. When *α* = 0.25, the average errors of 4-Multihop and i-Multihop are more than 30%, while those of MLGS and MLGS(R) are only less than 18%.

### 5.5. Impact of Grid Granularity on MLGS Algorithm

**Figure 18.**
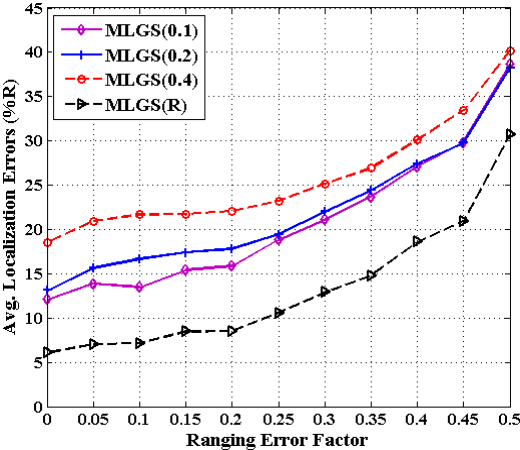
Average localization error *versus* grid granularity (isotropic network).

**Figure 19.**
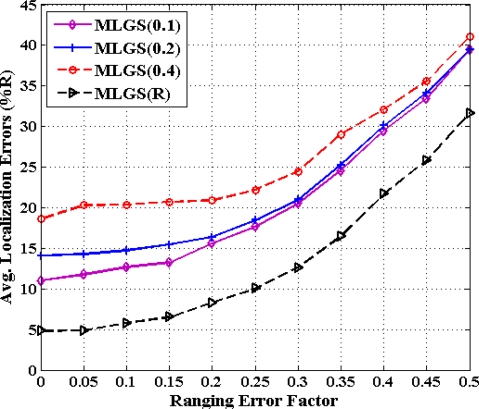
Average localization error *versus* grid granularity (anisotropic network).

From previous investigations, we draw a conclusion that MLGS produces better results in most cases. Here, we discuss the impact of grid granularity *g* on localization accuracy of MLGS under various ranging error factors (see Figures 18 and 19). In both figures, the decimals in the legends represent the values of grid granularity *g* for coordinate estimation. Generally, the localization accuracy of MLGS improves with grid granularity *g* declining. However, when *g* reduces to a certain extent, any further decrease of *g* does not yield any significant improvement in accuracy. In isotropic network, the average localization error with *α* = 0.1 can be reduced by 4.97% as *g* decreases from 0.4 to 0.2, while only 3.23% as *g* decreases from 0.2 to 0.1. In anisotropic network, the corresponding descents of average errors with *α* = 0.1 are 5.64% and 2.06%, respectively. In addition, with the increase of *α*, the impact of grid granularity *g* on localization accuracy drops gradually. For example, MLGS(0.1) and MLGS(0.2) nearly have the equivalent performance when *α* = 0.4. In the phase of coordinate estimation, there is *U* ∝ (1/*g*^2^), where *U* represents the number of samples. Thus, reducing *g* would make *U* grow significantly, which further leads to the increase of computation cost required in localization. The quantitative analysis of computation cost will be present in Section 5.6. In practice, we should determine the optimal grid granularity *g* based on the trade-off of localization accuracy and computation cost.

### 5.6. Comparisons of Computation Cost

**Figure 20.**
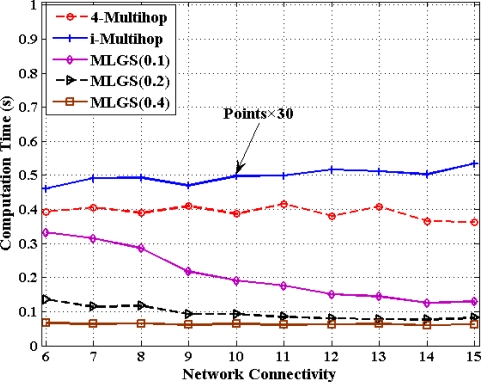
Computation cost *versus* network connectivity (isotropic network).

**Figure 21.**
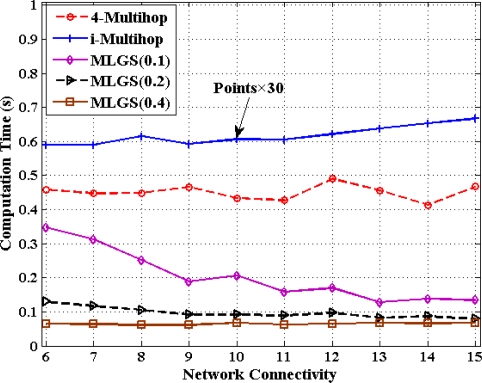
Computation cost *versus* network connectivity (anisotropic network).

In this part, we discuss the computation cost of 4-Multihop, i-Multihop and MLGS with the metric of total computation time for calculating the coordinates of all normal nodes under different degrees of network connectivity (see Figures 20 and 21). As i-Multihop employs the complex constrained nonlinear programming solver to estimate the coordinates of normal nodes, its computation cost is more than 30 times of those of 4-Multihop and MLGS. When network connectivity is low, the computation cost of MLGS(0.1) (no more than 0.35 s in both scenarios) is slightly smaller than that of 4-Multihop (0.4 s and 0.45 s in isotropic and anisotropic networks, respectively).

However, with the increase of network connectivity, MLGS(0.1) performs faster and faster, while 4-Multihop keeps a constant computation time. That is because higher network connectivity would enhance the constraints of sensor nodes and diminish the feasible regions of normal nodes in MLGS. For MLGS, reducing grid granularity *g* could lower the computation cost evidently in sparse networks. For example, in isotropic network with connectivity of 6, the computation time of MLGS(0.1) is 0.33 s, that of MLGS(0.2) is 0.14 s and that of MLGS(0.4) is only 0.06 s. But in networks with high connectivity, with the increase of *g*, the variety of computation cost is not so obvious. When network connectivity reaches 10, the computation cost of MLGS in different *g* drops to below 0.2 s. It is worth noting that MLGS(0.2) and MLGS(0.4) nearly have a constant computation time in various network connectivity.

### 5.7. Performance Comparisons of MLGS, MDS-MAP and Iterative Algorithms

**Figure 22.**
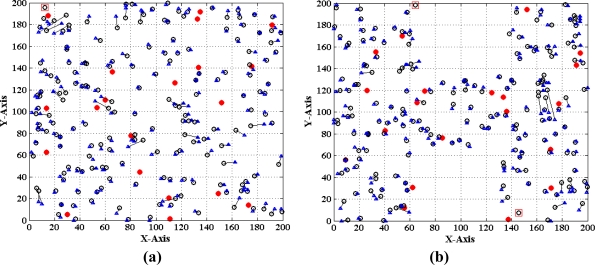
Localization results of MLGS. **(a)** Isotropic network. **(b)** Anisotropic network.

**Figure 23.**
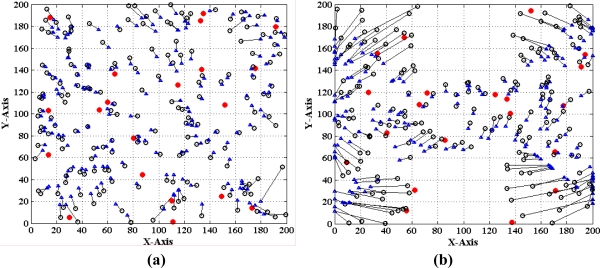
Localization results of MDS-MAP. **(a)** Isotropic network. **(b)** Anisotropic network.

**Figure 24.**
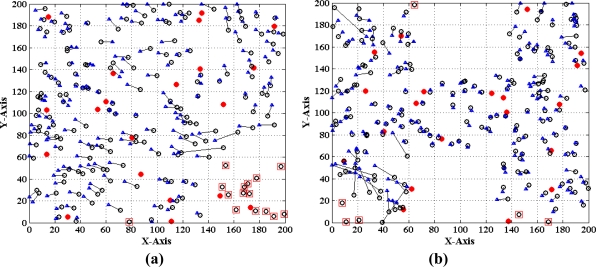
Localization results of iterative algorithm. **(a)** Isotropic network. **(b)** Anisotropic network.

Finally, we evaluate the performance of the MLGS by comparing it with MDS-MAP [7] and the improved iterative algorithm with error control mechanism similar to [14]. The comparison results are shown in Figures 22–24, in which the circles represent true positions of nodes (solid circles for anchor nodes and empty circles for normal nodes), the triangles represent estimation positions of normal nodes, and the lines represent localization errors. For MLGS and Iterative algorithms, if a normal node can’t get enough reference information to computing its coordinates, a square will be drawn around it. Table 2 gives the average localization errors of three algorithms in isotropic and anisotropic networks.

In isotropic network, MLGS has the best localization performance. Its average error is below 15% and the error distribution is uniform. One unlocalized node and a few normal nodes with bigger localization errors are mainly concentrated in the upper-left corner, where fewer anchor nodes exist. MDS-MAP has an average error of 23.1% and a localization coverage rate of 100%. The localization accuracies of edge nodes are worse than those of middle nodes. The iterative algorithm with average error of 25.8% and localization coverage rate of 91.7% performs the worst. The iterative process stops at the lower-right corner where sensor nodes are sparsely deployed. Furthermore, the impact of error accumulation is not totally eliminated in the improved iterative algorithm. As can be seen from the Figures 22 and 24, the localization accuracy of MLGS and iterative algorithm is not obviously affected by network topology. In anisotropic network, the average errors of both algorithms are 12.7% and 26.1%, respectively, which are close to those in isotropic network. However, the average error of MDS-MAP (62.1%) is much larger than that in isotropic network. That is because MDS-MAP needs to approximate the lengths of shortest paths to Euclidean distances between all pairs of non-neighboring nodes. Irregular network topology would make the approximation large errors, especially between pairwise nodes that are far apart.

## 6. Conclusions

In this paper, we present a novel multi-hop localization algorithm called MLGS, which is shown to be able to enhance the adaptability to irregular network topology, improve the positioning accuracy, as well as reduce computational cost for multi-hop localization in large-scale WSNs. We first analyze the factors that influence the multi-hop distance estimation and give a quantitative rule for setting the weight of reference information. Then, the close to optimal values of node coordinates are efficiently searched and obtained in the feasible regions of normal nodes through a lightweight grid-scanning scheme, which avoids solving the complex constrained nonlinear programming and prevents getting stuck at local optimum. MLGS is very suitable for sensor nodes of limited energy and computing power. Through extensive simulations in isotropic and anisotropy networks, we demonstrate that MLGS outperforms the typical multi-hop localization schemes in many aspects. Compared with MDS-MAP and iterative algorithm, MLGS can also do better in localization accuracy and topology adaptability. In most cases, MLGS could achieve better performance, even without refinement phase. Therefore, the phase of node collaboration refinement is optional. Reducing the grid granularity *g* in the phase of coordinate estimation can improve the localization accuracy of MLGS. However, when *g* reduces to a certain extent, the improvement of accuracy becomes more and more marginal as *g* further decreases. On the contrary, it raises the computation cost of sensor nodes. Empirically, MLGS could get good performance when the grid granularity *g* is set to 0.1∼0.2. In the future, we would like to extend MLGS to 3D WSNs and implement it on experimental WSNs prototypes to verify its practicability.

## Figures and Tables

**Table 1.** Default parameters of WSNs.

**Parameters**	**Isotropic network**	**Anisotropic network**
Network deployment area (m)	200 × 200	200 × 200
Network holes (m)	No apparent hole	66.7 × 66.7 (×2)
Number of nodes	200	200
TTL	5	5
Percentage of anchor nodes	10%	10%
Node’s communication radius (m)	25.6	24.2
Network connectivity	9	9
Ranging error factor	0.1	0.1

**Table 2.** Average errors of MLGS, MDS-MAP and iterative algorithms.

**Algorithms**	**Isotropic network**	**Anisotropic network**
MLGS	13.4%	12.7%
MDS-MAP	23.1%	62.1%
Iterative algorithm	25.8%	26.1%

**Figure A.**
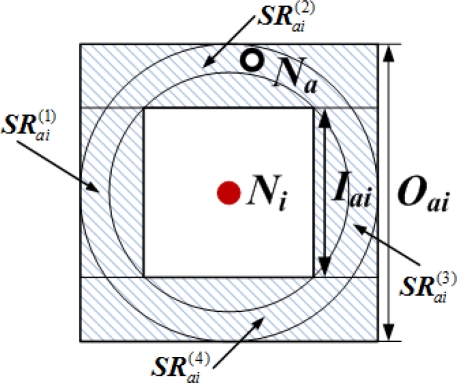
Division of bounding square ring.

**Figure B.**
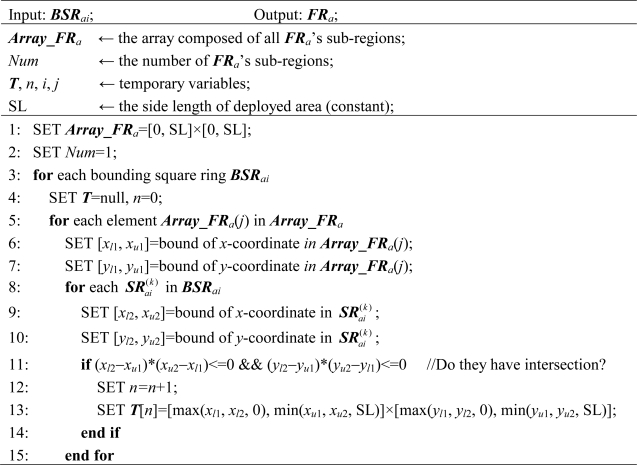

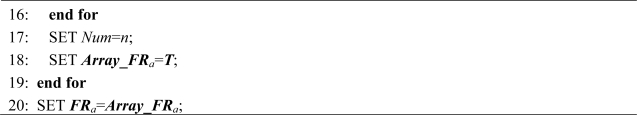
Pseudo-codes of determining ***FR****_a_*.

## Appendix

### Computation of Feasible Region *FR_a_*

As shown in Figure A, the bounding square ring ***BSR****_ai_* can be divided into four rectangular sub-regions, which are denoted as ${\mathrm{SR}}_{\mathrm{ai}}^{\left(k\right)}(k=1,2,3,4)$. Then, the feasible region ***FR****_a_* can be obtained through the procedure of which the pseudo-codes are shown in Figure B.
